# Validation of the Internal Structure of a German-Language Version of the Gender Role Conflict Scale – Short Form

**DOI:** 10.3389/fpsyg.2018.01161

**Published:** 2018-07-06

**Authors:** Nikola Komlenac, Heidi Siller, Harald R. Bliem, Margarethe Hochleitner

**Affiliations:** ^1^Gender Medicine Unit, Innsbruck Medical University, Innsbruck, Austria; ^2^Faculty of Psychology and Sport Science, University of Innsbruck, Innsbruck, Austria

**Keywords:** Gender Role Conflict Scale – Short Form, confirmatory factor analysis, Austrian men, Sexual Performance Belief Scale, validity, reliability

## Abstract

The Gender Role Conflict Scale – Short Form (GRCS-SF) assesses a person’s masculine gender role conflict. Masculine gender role conflict results when a person experiences discomfort showing a certain behavior because it is in conflict with masculine norms. The aim of the study was to test the questionnaire’s psychometric properties in an Austrian sample of older men. Three alternative structural models of the GRCS-SF were tested with confirmatory factor analyses (CFA). The maximum-likelihood method and the Bollen–Stine Bootstrap Method were used to estimate the fit indices of the CFA. Convergent validity was tested by correlating the GRCS-SF with the Sexual Performance Belief Scale (SPBS). Participating in the study were 127 male in-patients of a university hospital. Men’s average age was 59.5 (*SD* = 14.6) years. The one-factor model did not fit the empirical data well. In contrast, both the four-factor structure model and the bifactor structure model were supported. Good internal consistencies indicated acceptable reliabilities of the questionnaire’s scales. As expected, moderate to large correlations with the SPBS were detected. These findings support the claim that the GRCS-SF is a reliable and valid tool for assessing men’s gender role conflict also in a sample of older men in Austria.

## Introduction

O’Neil (2015) summarized findings from over 400 studies on gender role conflict (GRC), thereby illustrating the extent of research on GRC. GRC results when a person experiences stress or discomfort because the person has to decide between behaviors prescribed by masculine norms and other sets of behaviors that appear more adaptive in a given situation (O’Neil et al., 1986; Wester et al., 2012).

There are four patterns of masculine GRC (O’Neil et al., 1986). Success, power, and competition (SPC) refers to the constant obsession with being better and more successful than other people. The second pattern is called restrictive emotionality (RE). People who show RE refrain from expressing emotions. For men who show the pattern of restrictive affectionate behavior between men (RABBM), it is important to be perceived as heterosexual men by others. Therefore, they refrain from showing positive affection toward other men. The pattern conflict between work and family relations (CBWFR) pertains to men who are preoccupied with their work to such an extent that they do not find enough time for their family or other leisure actives. GRC has negative effects on oneself or others who are present in a certain situation (O’Neil, 2015).

To measure patterns of GRC O’Neil et al. (1986) developed the Gender Role Conflict Scale (GRCS). The psychometric properties of the GRCS have been tested in several samples in diverse countries. The GRCS proved to have a lack of factorial validity in some of these studies. Some authors suggested to remove items in order to improve the model fit and to make the scale more applicable to a culturally broader sample (O’Neil, 2015).

In order to respond to this demand, Wester et al. (2012) developed a shortened and more culturally applicable version of the GRCS, the Gender Role Conflict Scale – Short Form (GRCS-SF). However, both the original GRCS and the GRCS-SF were developed on the basis of samples that comprised undergraduate students. The GRCS has been criticized for using only such a sample. So far the psychometric properties of the GRCS-SF have been satisfactorily validated using the United States sample of community-dwelling and college men (Levant et al., 2015; Hammer et al., 2017) as well as a sample of Spanish college men (García-Sánchez et al., 2018). A further validation study of the GRCS-SF was conducted in China. In this case, a more diverse sample was selected among young men (Zhang et al., 2015).

The aim of the current analysis was to test the validity and reliability of a German-language version of the GRCS-SF. We tested the convergent validity by calculating the correlation between the Sexual Performance Belief Scale (SPBS) (Thompson and Barnes, 2013) and the GRCS-SF. Sexual performance can often serve as an outlet of performing and confirming one’s own masculinity (Lamb et al., 2018). Therefore, we hypothesized that men’s GRC would positively correlate with the SPBS.

We used a sample of men with diverse socio-demographic backgrounds. We thus add to the literature on the psychometric properties of the GRCS-SF and tested whether the claim of diverse cultural applicability of the GRCS-SF can be supported.

## Materials and Methods

### Participants

Full responses were provided by 127 in-patients (*M*_age_ = 59.5, *SD* = 14.6, range = 23–84) at a university hospital in Austria. Most participants (88.2%) indicated having Austrian nationality. Additional nationalities reported were: German (6.3%), Italian (2.4%), Turkish (0.8%), or other (2.4%). The majority of men were retired (53.5%). Of the men, 41.7% were employed and 1.6% were in vocational training, whereas 3.1% were neither employed nor in vocational training. Most men (91.3%) self-reported having a heterosexual sexual orientation. Of the participants, 1.6% had a homosexual sexual orientation, 1.6% had a bisexual sexual orientation, and 2.4% had an asexual sexual orientation (3.1% unidentified sexual orientation).

### Measures

The GRCS-SF (Wester et al., 2012) measures four patterns of GRC as presented in the Section “Introduction.” The questionnaire consists of 16 items, whereby four items are dedicated to each of these patterns of GRC. Men were asked to indicate the degree of experienced conflict on a six-point Likert’s scale ranging from 0 (*strongly disagree*) to 5 (*strongly agree*). Higher scores were an indication of GRC. Wester et al. (2012) reported satisfactory internal consistencies (α = 0.77–0.80). For the Austrian sample, the GRCS-SF was translated into German by the first author. After he translated the questionnaire, he read the translation several times. The second author checked the translation and marked points she disagreed with. These were resolved through discussion and consensus.

The Sexual Performance Belief Scale (SPBS) (Thompson and Barnes, 2013) assesses whether men believe in traditional masculine norms concerning “sexual performance.” The questionnaire asks whether men think a man is supposed to have high sexual desire, needs to achieve a rigid erection or that partnered sexual activity is a “performance” in order to not lose “masculinity.” Men were asked to indicate the degree of agreement with such traditional masculine norms about sexuality on a seven-point Likert’s scale ranging from 0 (*strongly disagree*) to 6 (*strongly agree*). The SPBS is reported to have satisfactory internal consistencies of Cronbach’s α = 0.82 (Thompson and Barnes, 2013). In the current study, the same internal consistency was calculated. The SPBS was translated into German using the same approach used for the GRCS-SF.

### Procedure

Data collection for this study occurred as part of a larger research project (Komlenac et al., 2018). The study was approved by the Innsbruck Medical University Hospital’s Ethics Committee (ID: AN2016-0093 362/4.5). The study was conducted in accordance with the Declaration of Helsinki (World Medical Association, 2013). The inclusion criteria for participants were male gender, age over 18 years and ability to speak and understand German. All patients were given verbal and written information about the study. Written informed consent was obtained from all participants who agreed to participate. No reimbursement was offered.

### Statistical Analysis

The data were analyzed with R (R Core Team, 2018), version 3.5.0, using the *MBESS* package (Kelley, 2018) and the *psych* package (Revelle, 2018). The CFA was calculated with IBM SPSS Amos, version 24.0 (Arbuckle, 2016). The level of significance for all analyses was α = 0.050.

### Testing the Models

For estimating the model fit of the CFA, maximum-likelihood estimations were calculated. Because of the violation of normal distribution and because of a violation of multivariate normality the Bollen–Stine bootstraps (Bollen and Stine, 1992) with 500 bootstrap samples were used to estimate chi-square as a model fit index (Weiber and Mühlhaus, 2014). Further, we adjusted the estimates of the fit indices, namely the root mean square error of approximation (RMSEA) and the comparative fit index (CFI), to account for the violation of normality as explained by Walker and Smith (2017). Significant *p*-values indicated an inadequate fit of the model and the empirical data. A good model was assumed when RMSEA did not exceed the value of 0.06 and the standardized root mean square residual (SRMR) did not exceed the value of 0.08 (Hu and Bentler, 1999). The CFI was expected to be equal to or higher than the value of 0.90 (Weiber and Mühlhaus, 2014). To test the convergent validity, Spearman’s correlations between the GRCS-SF scales and the SPBS were calculated.

Three competing models were calculated. The first model tested was a one-factor model that comprised only one factor that would explain all 16 items of the GRCS-SF. Second, the original four-factor model (Wester et al., 2012) was tested. Lastly, a bifactor model was tested. In this last model, each item loaded directly on one general factor in addition to one of the four specific factors that are in accordance with the four patterns of GRC (Chen et al., 2012).

## Results

### Confirmatory Factor Analysis

The one-factor model proved to have the least satisfying fit indices. Even though the descriptive fit indices were satisfactory, the significant chi-test indicated that the one-factor model was an inadequate fit to the data (**Table 1**).

**Table 1:** Goodness-of-fit indices of the models (*N* = 127).

Model	Fit indices				
	χ^2†^	df	CFI^†^	RMSEA^†^	SRMR
Single factor	150.38^∗∗^	104	0.91	0.06	0.11
Four factors	114.19	98	0.97	0.04	0.08
Bi-factor	96.07	88	0.99	0.03	0.06

*df = degrees of freedom; RMSEA = root mean square error of approximation, SRMR = standardized root mean square residual; CFI = comparative fit index; CI = confidence interval. ^†^Adjusted indices with the method offered by Walker and Smith (2017). ^∗∗^p < 0.010.*

The original four-factor model and the bifactor model proved to be an acceptable fit to the data. Non-significant chi-tests indicated that the models did not differ from the empirical data. The RMSEA and SRMR did not exceed preferred values in either model and the CFI was higher than 0.90 (**Table 1**).

The four-factor model was supported by items’ significant loadings to the respective factor (λ = 0.33–0.83; **Table 2**). Structure coefficients offered further evidence that the items loaded high on the respective factor whereby having low factor loadings on each of the other three factors (**Table 2**). Tucker’s congruence coefficients (Tucker, 1951) were calculated to judge the similarities of the current four-factor model’s factor loadings and those factor loadings obtained by Wester et al. (2012). Tucker’s congruence coefficients were all higher than 0.95 (φ = 0.96–0.99). Such coefficients indicate good similarities between the compared factors (Lorenzo-Seva and Ten Berge, 2006). In the four-factor model, all four scales correlated with each other to a moderate extent (*r* = 0.36–0.44) (Cohen, 1988).

**Table 2:** Means, McDonald’s ω, factor loadings in the confirmatory factor analysis, and Spearman’s correlation (*N* = 127).

Factor and items	*M* (*SD*)	ω (95% CI)	Standardized factor loadings (Four-factor model)	Structure coefficients (4-factor model)				Item-test correlation	Correlation with the SPBS^a^
				CBWFR	RE	RABBM	SPC		
CBWFR	2.2 (1.2)	0.68 (0.57–0.75)							0.18^∗^
Item 1	2.0 (1.6)		0.33***	0.33	0.14	0.12	0.15	0.36***	
Item 10	2.2 (1.7)		0.82***	0.82	0.35	0.29	0.36	0.58***	
Item 13	2.0 (1.6)		0.68***	0.68	0.29	0.24	0.30	0.55***	
Item 16	2.8 (1.7)		0.48***	0.48	0.20	0.17	0.21	0.38***	
RE	1.6 (1.1)	0.75 (0.65–0.82)							0.32**
Item 5	1.4 (1.4)		0.78***	0.33	0.78	0.31	0.33	0.55***	
Item 6	1.4 (1.5)		0.83***	0.35	0.83	0.33	0.35	0.58***	
Item 8	1.3 (1.4)		0.62***	0.26	0.62	0.25	0.26	0.50***	
Item 12	2.3 (1.5)		0.37***	0.16	0.37	0.15	0.16	0.48***	
RABBM	2.0 (1.4)	0.78 (0.70–0.84)							0.37**
Item 3	1.6 (1.8)		0.57***	0.21	0.23	0.57	0.21	0.52***	
Item 7	2.5 (2.0)		0.79***	0.28	0.32	0.79	0.29	0.62***	
Item 9	2.1 (1.9)		0.72***	0.26	0.29	0.72	0.26	0.61***	
Item 14	1.7 (1.6)		0.65***	0.23	0.26	0.65	0.24	0.55***	
SPC	1.8 (1.1)	0.73 (0.64–0.80)							0.42**
Item 2	2.4 (1.6)		0.47***	0.21	0.20	0.17	0.47	0.45***	
Item 4	1.6 (1.4)		0.55***	0.24	0.23	0.20	0.55	0.61***	
Item 11	1.9 (1.7)		0.77***	0.34	0.32	0.28	0.77	0.55***	
Item 15	1.4 (1.3)		0.77***	0.34	0.33	0.28	0.77	0.53***	

^a^Spearman’s correlation with N = 124. SPBS = Sexual Performance Belief Scale; CBWFR = Conflict Between Work and Family Relations; RE = Restrictive Emotionality; RABBM = Restrictive Affectionate Behavior Between Men; SPC = Success/Power/Competition. *p < 0.050, **p < 0.010, ***p < 0.001.

### Reliability and Convergent Validity

All four scales of the GRCS-SF had acceptable internal consistencies (McDonald’s ω = 0.68–0.78; **Table 2**) (McDonald, 1999). Furthermore, all items had moderate to high item-test correlations (*r* = 0.36–0.61; see **Table 2**) and the hierarchical ω was ω_h_ = 0.47 (McDonald, 1999).

There were significant positive small to moderate correlations between all four scales of the GRCS-SF and the SPBS (*r* = 0.18–0.42; **Table 2**) (Cohen, 1988). The scale SPC was most associated with believing in the masculine norm concerning sexual performance.

## Discussion

The main aim of the current analysis was to validate the psychometric properties of a German-language version of the GRCS-SF. We found a good fit for the four-factor as well as the bifactor structure of the GRCS-SF. These results are comparable to those of other studies that analyzed the psychometric properties of the GRCS-SF (Levant et al., 2015; Zhang et al., 2015; Hammer et al., 2017; García-Sánchez et al., 2018). Additionally, the factor loadings of the four-factor model are very similar to those obtained in the original study by Wester et al. (2012). The one-factor solution, on the other hand, was the worst fitting model. Therefore, the conclusion can be supported that the GRCS-SF assesses four different patterns of a common construct, e.g., the GRC (Levant et al., 2015).

The GRCS-SF was developed and mostly tested on samples comprising college men or young men. However, Wester et al. (2012) developed the GRCS-SF with the intention that it would be used in more culturally and socio-demographically diverse samples. The current analysis tested the psychometric properties in an Austrian sample of men with diverse socio-demographic background who were older than men in previous studies of the GRCS-SF’s psychometric properties (Levant et al., 2015; Zhang et al., 2015; García-Sánchez et al., 2018). Therefore, the results of the current analysis support the claim that the GRCS-SF is applicable in samples of men with diverse socio-demographic backgrounds.

This analysis showed the GRCS-SF’s convergent validity with the SPBS (Thompson and Barnes, 2013). Especially, the scale measuring men’s attitudes toward competition, success, and power was associated with their beliefs that partnered sexual activity is an outlet that men need to “perform” successfully in order to prove their masculinity. Thus, the convergent validity of the GRCS-SF with a related construct was evident.

The study has its limitations. The small sample size or the specificities of an in-patient sample limit the generalizability of the findings. Additionally, the usage of maximum-likelihood estimations on data that violate multivariate normality can bias results and limit the generalizability. However, we tried to account for this limitation by adjusting the fit indices with the Bollen–Stine bootstraps (Bollen and Stine, 1992) as suggested by Walker and Smith (2017).

Further validation studies of the German-language version of the GRCS-SF are needed. Nevertheless, this is the first study to analyze the GRCS-SF in a sample of older men with diverse socio-demographic background in Austria. It adds findings that recommend the use of the GRCS-SF to reliably and validly assess men’s GRC.

## Availability of Data and Materials

The datasets used and/or analyzed during the current study are available from the corresponding author on reasonable request.

## Ethics Statement

The study was conducted in accordance with the Declaration of Helsinki (World Medical Association, 2013). The study was approved by the Innsbruck Medical University Hospital’s Ethics Committee (ID: AN2016-0093 362/4.5). All subjects gave written informed consent in accordance with the Declaration of Helsinki.

## Author Contributions

NK, HS, HB, and MH designed the research. NK collected the data. NK wrote the manuscript. HS and NK participated in the data analysis. NK, HS, HB, and MH discussed the current results in relation to the literature. All the authors read and approved the final manuscript.

## Conflict of Interest Statement

The authors declare that the research was conducted in the absence of any commercial or financial relationships that could be construed as a potential conflict of interest.
